# Comparison of outpatient health care utilization among returning women and men Veterans from Afghanistan and Iraq

**DOI:** 10.1186/1472-6963-10-175

**Published:** 2010-06-22

**Authors:** Mona Duggal, Joseph L Goulet, Julie Womack, Kirsha Gordon, Kristin Mattocks, Sally G Haskell, Amy C Justice, Cynthia A Brandt

**Affiliations:** 1 Department of Internal Medicine, Yale University School of Medicine, New Haven, CT, USA; 2 Center for Medical Informatics, Yale University, New Haven, CT, New Haven, CT, USA; 3 VA Connecticut Health Care System, 950 Campbell Ave, West Haven, CT, USA

## Abstract

**Background:**

The number of women serving in the United States military increased during Operation Enduring Freedom (OEF) and Operation Iraqi Freedom (OIF), leading to a subsequent surge in new women Veterans seeking health care services from the Veterans Administration (VA). The objective of this study was to examine gender differences among OEF/OIF Veterans in utilization of VA outpatient health care services.

**Methods:**

Our retrospective cohort consisted of 1,620 OEF/OIF Veterans (240 women and 1380 men) who enrolled for outpatient healthcare at a single VA facility. We collected demographic data and information on military service and VA utilization from VA electronic medical records. To assess gender differences we used two models: use versus nonuse of services (logistic regression) and intensity of use among users (negative binomial regression).

**Results:**

In our sample, women were more likely to be younger, single, and non-white than men. Women were more likely to utilize outpatient care services (odds ratio [OR] = 1.47, 95% confidence interval [CI]:1.09, 1.98), but once care was initiated, frequency of visits over time (intensity) did not differ by gender (incident rate ratio [IRR] = 1.07; 95% CI: 0.90, 1.27).

**Conclusion:**

Recently discharged OEF/OIF women Veterans were more likely to seek VA health care than men Veterans. But the intensity of use was similar between women and men VA care users. As more women use VA health care, prospective studies exploring gender differences in types of services utilized, health outcomes, and factors associated with satisfaction will be required.

## Background

Of the over 900,000 service members who have returned from active duty during Operation Enduring Freedom (OEF) and Operation Iraqi Freedom (OIF), 102,000 (11%) are women [1,2]. Women currently constitute a higher proportion of military service members than at any other time in United States history [2]. Compared with Veterans of prior conflicts, OEF/OIF Veterans are seeking Veterans Administration (VA) medical services in greater numbers [3]. The current demand for VA medical services has far exceeded what had been anticipated [4]. An influx of OEF/OIF women Veterans will likely create increased demand for VA health care services beyond the levels usually associated with the VA.

Gender differences in health care use are a combination of interacting economic, social, psychological and biological factors. Although, men aged more than ≥ 59 years have higher health care utilization than women of the same age, younger women report higher use of health care services than men during their child bearing years [5,6]. These differences may be associated with reproductive biology, gender-specific conditions and higher morbidity rates in women in this age group [5,7-10]. Approximately 85% of OEF/OIF women Veterans are under 40 years [11]. In addition, other socio-demographic characteristics such as being single, low income and education levels, and belonging to a minority group have been found to negatively affect health care utilization [12-15]. Therefore understanding the effect of gender and other underlying determinants on use of health care services is important in promoting equal access to health care.

OEF/OIF women Veterans' use of VA health services is a relatively new area of research. Few published peer-reviewed studies to date have examined the association between gender and health care service utilization rates among OEF/OIF Veterans. In previous eras of military service, women Veterans were much less likely to use VA services than their male counterparts, with differences between men and women in seeking VA health care services attributed to biases in the provision of care for women Veterans [2,16,17]. Overall, however, more than 40% of men and women OEF/OIF Veterans have enrolled for VA health care services [1,2]. Due to this surge in new women Veterans seeking health care services from the VA, genderdifferences in utilization of VA outpatient care clinics may determine if there are any gender-related disparities in utilization. Therefore, the objective of this study was to examine gender differences in utilization of outpatient VA health care services among OEF/OIF Veterans at a single VA facility. We hypothesized that among OEF/OIF Veterans, women have higher utilization of outpatient VA health care services than men. Our findings expand upon the current literature on health care services utilization among Veterans by examining gender differences in use and intensity of use of outpatient healthcare services of enrolled OEF/OIF Veterans.

## Methods

The institutional review boards at the local Veterans Affairs Medical Center (VAMC) and Yale University approved this study.

### Sample

Our initial sample consisted of 1820 OEF/OIF Veterans who enrolled for care at the local VAMC or at one of the six associated community-based outpatient clinics (CBOCS) between September 1, 2002, and October 30, 2006. The OEF/OIF manager at the local facility provided a roster of enrolled OEF/OIF Veterans. However, the addresses of many of these Veterans (as determined by zip codes) were outside the state or were within the vicinity of other VA medical facilities. Since one of the aims of this study was to explore intensity of use of services, we included only Veterans living in zip codes within a 100-mile radius of our facility and its associated CBOCS. By restricting the sample, we also sought to control for the effect of distance on utilization intensity [18,19]. Using this criterion, we excluded 200 Veterans, making our final sample size 1620 Veterans.

### Data Sources

Information on socio-demographic characteristics and health care utilization was abstracted from the VA electronic medical record (EMR) system. Military service variables were obtained from the local OEF/OIF roster.

### Outcome Variables

Based on our primary objective, we operationalized outpatient healthcare use in a sequential two-step process to outpatient care visit resulting in two related measures. We defined a visit as a face-to-face outpatient encounter on a unique date and location. The outpatient VA services use include basic care (e.g., primary care, mental health) specialty care (e.g. services by clinical specialist such as surgeon, radiologist, cardiologist etc.) and other ancillary care services such as preventive screening, immunization, visits to outpatient pharmacy, counseling sessions for weight, smoking etc, labs and electrocardiograms [20]. The first measure, initiation of care, was defined as having at least one visit to the outpatient care clinic. Only visits after return from OEF/OIF deployment were included. The second measure, intensity of utilization, was described as the number of visits, including the initial visit, among those who used outpatient care during the study period. This approach recognizes differences in the processes affecting initiation of care and rate of health care use [21,22].

### Model Covariates

Gender was the primary predictor variable. Additional covariates consisted of socio-demographic and military service related characteristics, including age, race (white, non-white, unknown), marital status (married, single), presence of private health insurance, length of military service (< 3 years vs. ≥3 years), service branch (e.g. Army, Marine Corps), and percent service-connected disability (< 30% vs. ≥30%). Age was included as a continuous variable. Service-connected disability, a known predictor of use of health care services within the VA system, was defined as an injury or illness that either was incurred or aggravated by military service affecting employability or functioning [23,24]. This is based on the proportion of the disability assigned by Veterans Benefits Administration [3,25,26]. Persons with more than 30% service connected disability are entitled to maximum benefits, including Veterans' retirement pay, disability severance pay, separation incentive payments, and increased amounts of VA compensation [25,27]. Therefore, we dichotomized this variable to individuals with ≥30% and < 30% service connected disability, including no disability.

### Data Analysis

Socio-demographic and military characteristics were compared with utilization status and gender using the chi-squared test for categorical variables and the t test for continuous measures to determine the association between the independent variables and the outcomes of interest. We also compared differences between female users and non-users and male users and non-users. We utilized logistic regression analysis to compare the odds of initiating outpatient care among women and men and standard negative binomial regression analysis to assess the intensity of utilization. A negative binomial model is preferred over Poisson regression for count data when data are over-dispersed (i.e., the variance is greater than the mean), as in our study [26,28]. Both the logistic and negative binomial models included the same covariates that have been shown to have an impact on both initiation and intensity. To adjust for the varying length of observation time per subject, we incorporated the log of the time variable as an offset in the negative binomial model. We also included interaction terms between gender and other covariates of interest to explore the extent to which significant predictors moderated any between-gender differences identified. A significant interaction (P < 0.05) would indicate that the association of gender with the outcome varied at levels of the other variable. All statistical analyses were performed using Stata/SE 9.2 for Windows (Stata Corporation, College Station, TX, USA).

## Results

We identified 240 women and 1380 men OEF/OIF Veterans who met the eligibility criteria for this study. The mean age of the study population was 33 years (Table 1), with approximately 60% each being single, without any outside health insurance, and self identifying as being of white race. Over 60% had served in the military for less than 3 years, with the majority (70%) having served in the Army. During the observation period, 54% (874/1620) of the study population did not have any visits to the local VAMC for outpatient health care. Bivariate analyses showed that users and non-users of medical services did not differ significantly in age (p = 0.30), or length (p = 0.84) or branch (p = 0.23) of service. We observed a significant difference in utilization by gender, in that women were significantly more likely than men to have utilized care [p = .01].

**Table 1:** Sample Characteristics by Health Care Utilization Status

	Overall N = 1620	Users N = 746	Non Users N = 874	P value
**Sex**				
Women	15	17	12	0.14
Men	85	83	87	
**Mean Age (S.D.)**	33(9.13)	34(9.48)	33(8.41)	0.01
**Race (%)**				
White	59	65	54	
Non White	11	13	9	0.001
Unknown	30	22	37	
**Marital Status (%)**				
Married	37	38	35	
Single	63	62	65	0.3
**Length of Service (%)**				
< 3 years	66	67	66	0.84
≥3 years	34	35	34	
**Have Private Health Insurance (%)**	41	48	35	0.001
**Having ≥30% Service Connected Disability (%)**	12	17	7	0.001
**Service Branch (%)**				
Army				
Navy	70	71	68	
Air force	8	8	9	0.23
Marine Corps	8	8	8	

Women non users compared to men non users (Table 2) were significantly younger(mean age 29 vs. 33 years, p < 0.001) and were more likely to be from the army service branch (75% vs. 68%, p = 0.04). There was no significant difference by race (p = 0.7), marital status(p = 0.13) having private health insurance (p = 0.6), a service-connected disability (p = 0.7) or length of service (p = 0.3).

**Table 2:** Sample Characteristics of Veterans Not Using Care by Gender

Characteristics	Men N = 762	Women N = 112	P Value
**Mean Age (S.D.)**	33(8.60)	29(6.28)	0. 0001
**Race (%)**			
White	55	50	
Non White	8	12	0.42
Unknown	37	38	
**Marital Status (%)**			
Married	36	29	0.13
Single	64	71	
			
**Length of Service (%)**			
< 3 years	66	62	0.30
≥3 years	34	37	
**Have Private Health Insurance (%)**	36	39	0.60
**Having ≥30% Service Connected Disability (%)**	7	7	0.7
**Service Branch (%)**			
Army	68	75	
Navy	8	11	
Air force	7	8	0.04
Marine Corps	17	6	

Among the individuals who utilized VA healthcare (746/1620), more women utilized care than men (53% vs. 45%, p < 0.01). Women health care users were more likely than men health care users to be younger (Table 3) (mean age 31 vs. 34 years, p < 0.001), single (74% vs. 62 %, p < 0.001) and non-white (24% vs. 12%, p < 0.001). Men and women did not differ in having private health insurance (p = 0.7), a service-connected disability (p = 0.8) or length of service (p = 0.2). Women and men users showed no differences in the mean ± standard deviation (SD) (9.7 ± 10.6 vs. 9.47 ± 9.79, p = 0.8) and median (6.5 versus 6.0, p = 0.6) number of outpatient visits (Table 3). The range of study time was between 0.5 years and 2.3 years for the visits. The average observation time for women and men was similar (0.71 ± 0.61 vs. 0.79 ± 0.63 years, p = 0.22). The average number of outpatient visits per person per year, for women and men was 16.3 and 15.4 visits per year, respectively (p = 0.7).

**Table 3:** Sample Characteristics of Veterans Utilizing Care by Gender

Characteristics	Veterans Using Care		
	Men N = 618	Women N = 128	P Value
**Mean Age (S.D.)**	34(9.72)	31(7.72)	0.001
**Race (%)**			
White	67	53	0.01
Non White	12	24	
Unknown	21	23	
**Marital Status (%)**			
Married	38	26	0.01
Single	62	74	
			
**Length of Service (%)**			
< 3 years	67	62	0.2
≥3 years	33	38	
**Have Private Health Insurance (%)**	48	46	0.7
**Having ≥30% Service Connected Disability (%)**	17	15	0.8
**Service Branch (%)**			
Army	71	73	
Navy	7	10	0.01
Air force	7	10	
Marine Corps	15	7	
			
**Outpatient Visits**			
Mean(S.D.)	9.7(10.6)	9.5(9.8)	0.8
Median	15.4	16.3	0.7
Range(Min-Max)	1-55	1-56	
Mean Observation Time (Year)(S.D.)	0.80(0.63)	0.71(0.61)	0.22

### Results of initiation of care analysis

Multivariable logistic regression analysis showed that, after adjusting for age, race, marital status, private health insurance, service-connected disability, length of service and type of service branch, women were more likely to initiate care than men (OR = 1.47; 95% CI: 1.09, 1.98) (Table 4). Other characteristics associated with initiation of care included having a ≥30% service connected disability (OR = 2.73; 95% CI: 1.93, 3.85) and having private health insurance (OR = 1.44; 95% CI: 1.15, 1.86). For the logistic regression interaction model, the following interaction terms were included: sex*race, sex*marital status, sex*covered by health insurance, sex* service connected disability. No interaction terms were significant so they were omitted from the final model.

**Table 4:** Adjusted Logistic and Negative Binomial Regression Models for Outpatient Care Use

Variables	OR (95%CI)*	P value	IRR(95% CI)**	P value
	**(N = 1620)**		**(N = 746)**	
**Sex**				
Men(Ref)	1.00		1.00	
Women	1.47(1.09-1.98)	0.01	1.07(0.90-1.27)	0.4
				
**Race**				
White(ref)	1.00		1.00	
Non white	1.22(0.87-1.71)	0.26	0.90(0.75-1.09)	0.26
Unknown	0.61(0.5-0.78)	0.001	0.80(0.67-0.93)	0.01
				
**Mean Age**	1.02(1.-1.03)	0.01	1.01(0.99-1.01)	0.13
				
**Marital Status**				
Married(Ref)	1.00		1.00	
Single	1.15(0.89-1.46)	0.8	0.87(0.74-0.98)	0.05
				
**Length of service**				
<3 years	1.00		1.00	
≥ 3 years	1.00(0.80-1.24)	0.9	1.14(1.00-1.30)	0.05
				
**Have Private Health Insurance**	1.44(1.15-1.80)	0.002	0.77(0.67-0.88)	0.001
				
**Having Service Connected Disability**				
<30%(Ref)	1.00		1.00	
≥30%	2.73(1.93-3.85)	0.001	1.03(0.88-1.21)	0.69
				
**Service Branch**				
Army(Ref)	1.00		1.00	
Navy	0.79(0.54-1.16)	0.3	0.82(0.65-1.03)	0.09
Air force	1.00(0.67-1.50)	0.9	1.52(1.14-2.02	0.004
Marine Corps	0.92(0.67-1.27)	0.64	1.14(0.92-1.41)	0.24

*Logistic regression model for any use el for vs. no use **Negative binomial regression model for rate

### Results of rate of care analysis

In the adjusted negative binomial regression model, the intensity of utilization among health services users was similar for women and men (incident rate ratio (IRR) = 1.07; 95% CI: 0.90, 1.27) (Table 4). The rate did not differ by race (IRR = 0.90; 95% CI: 0.75, 1.09) or by percentage of service-connected disability (IRR = 1.03; 95% CI: 0.88, 1.21). Veterans with longer length of service(≥ 3 years) had significantly higher intensity of use (IRR = 1.14; 95% CI: 1.00-1.30). Veterans with health insurance were less likely than those without insurance to continue utilizing outpatient care at the VA (IRR = 0.77; 95% CI: 0.67, 0.88). The interaction model investigated the interactions between sex and age, marital status, race, and service branch, but none of these interaction terms was significant and they were omitted.

## Discussion

We have demonstrated that returning OEF/OIF women Veterans who were eligible for VA care were more likely than men to initiate use of health care services at VA facilities. However, there was no gender difference in the intensity of use, even after adjusting for socio-demographic and military service variables. Our study has some important strengths and limitations. While, this analysis is reflective of only those Veterans who sought health care at a specific VA facility and may not reflect OEF/OIF Veterans in other VA networks or those using services outside the VA, the results of our study support our hypothesis that OEF/OIF women Veterans were more likely to seek VA care than men Veterans. Although intensity of use was similar between women and men users of VA care, insufficient power may explain why the intensity of utilization and interaction terms were insignificant. Additionally, because of the small number of women included in our study population, we combined utilization at VA facilities with that at CBOCSs to increase the sample size. This site effect may have altered our findings, as initiation and utilization rates may differ between the VA Medical Center and the CBOCS. Recent studies have shown differences in the overall characteristics of Veterans utilizing CBOCS and VA Medical Centers [25]. A larger representative sample of OEF/OIF Veterans visiting other VA healthcare facilities is therefore required to better explore gender differences in health care utilization. Further analysis on types of services utilized over time and factors influencing satisfaction are required to better inform new policy decisions or recommendations. Finally, despite not having data on several important variables, such as socioeconomic status, education, and provider characteristics, the main strength of this study is that we had complete information on selected independent predictors of health care utilization variables for both users and non-users, as well as assessing sufficient numbers of women and men to allow for meaningful comparisons [26,28-30].

Our primary finding, that enrolled women OEF/OIF Veterans were more likely than their male counterparts to initiate care in the VA healthcare system, a significant change from earlier conflicts, should be interpreted in the context of continuous efforts by the VA over the past two decades to provide quality health care to women [30]. It has been estimated that only 14% of women Veterans of the Persian Gulf War (1990-1991) used VA healthcare after returning [18]. In the 1990 s, to meet the needs of this rapidly growing population, the VA adopted new models for delivering gender-specific health care services. This included the creation of separate women's health clinics at tertiary VA facilities, access to one or more female providers, and provision of basic gender-specific services, including pelvic examinations [31,32]. Nevertheless, lack of information about available VA services was cited as one of the most important barriers to women's use of VA healthcare [33]. In response, the VA implemented outreach and personal assistance initiatives specifically aimed at women [34,35]. Women at our site possibly were aware of a separate women's clinic at the Veterans Administration Medical Center and therefore may have been more likely to initiate care. Although we did not directly assess the impact of these efforts on utilization, our results suggest that these steps could have contributed to the increase we observed in the decision by women OEF/OIF Veterans to initiate health care use. An additional factor contributing to the increased use of health services by women Veterans may have been that, compared with previous conflicts, more OEF/OIF women had been exposed to combat and thus had higher levels of service-connected disability [36]. Our results confirm this finding, as female users had higher levels of service-connected disabilities than male users.

Conversely, however, the higher probability of initiation of care by women did not extend to increased intensity of use. This finding differs from the results of recent studies on gender and VA utilization, which showed higher outpatient health care utilization rates by women who were deployed or worked in non-traditional occupations [36,37]. These divergent results may be due to women using non-VA care for gender specific services that are not provided by the VA. Many VA outpatient clinics and physicians, especially those at the CBOCSs, are unable to provide basic healthcare services for women such as reproductive care, including pregnancy-related care, contraception, and care related to menopause [38]. The availability of health care providers and services plays a major role in decisions to continue utilizing care after enrollment. Since most of the OEF/OIF women Veterans in our study population were less than 40 years of age, they may have utilized outside care for reproductive health-related services, which are often contracted out by the VA healthcare system to local community providers or affiliates. Women in general often use their obstetrics and gynecology provider as their primary care provider. We did not have records of outside visits and therefore could not assess the intensity of health care use outside the VA [2,38,39].

## Conclusion

Our results suggest that among recently discharged Veterans women were more likely to seek VA care than men Veterans, indicating the need for comprehensive gender-specific outpatient care services for women within the VA healthcare system. Additional information on the organization, structure, and management of women's health practices, types of services utilized over time, health outcomes, factors influencing satisfaction with health care, and utilization costs is required to improve health care delivery to women at VA facilities. With the increase in the number of OEF/OIF Veterans using VA health care, understanding gender differences in their utilization of health care services may help VA policy makers provide better care to both women and men.

## Competing interests

The authors declare that they have no competing interests.

## Authors' contributions

MD was involved in the conception or design of the study, drafted the manuscript, and performed the statistical analysis. JG supervised the statistical analysis and data interpretation, and made substantive contributions to the manuscript. JW has revised the manuscript critically and made substantial contributions to the manuscript CB, SH and AJ revised the manuscript critically and made substantial contributions to conception and design of the present study, and acquisition of data. KG and KM reviewed the paper, discussed the results and implications, and commented on the manuscript at all stages All the authors have read and approved the final manuscript.

## Pre-publication history

The pre-publication history for this paper can be accessed here:

http://www.biomedcentral.com/1472-6963/10/175/prepub

## References

[B1] http://www.pdhealth.mil/education/2009_presentations/Dod_VA_State_Community_OEF_OIF_Partnership.pdf http://www.charlotteahec.org/ncahecswc2009/handouts/training_providers_to_work_veterans.ppt

[B2] www.hsrd.research.va.gov http://www.hsrd.research.va.gov/meetings/QUERI08/downloads/1017-Yano-2.ppt

[B3] http://www.academyhealth.org http://www.academyhealth.org/files/2008/monday/tmarshalls/6_9_2008_11_30/deytonl.pdf

[B4] Blimes L Soldiers Returning from Iraq and Afghanistan: The Long-term Costs of Providing Veterans Medical Care and Disability Benefits KSG Faculty Research Working Paper Series 2007 RWP07-001 1 20

[B5] Mustard CA Kaufert P Kozyrskyj A Mayer T Sex differences in the use of health care services N Engl J Med 1998 338 23 1678 1683 10.1056/NEJM199806043382307 9614260

[B6] Kjerulff KH Frick KD Rhoades JA Hollenbeak CS The cost of being a woman: a national study of health care utilization and expenditures for female-specific conditions Womens Health Issues 2007 17 1 13 21 10.1016/j.whi.2006.11.004 17321943

[B7] Verbrugge LM Work satisfaction and physical health J Community Health 1982 7 4 262 283 10.1007/BF01318959 7130446

[B8] Verbrugge LM Gender and health: an update on hypotheses and evidence J Health Soc Behav 1985 26 3 156 182 10.2307/2136750 3905939

[B9] Gijsbers van Wijk CM van Vliet KP Kolk AM Everaerd WT Symptom sensitivity and sex differences in physical morbidity: a review of health surveys in the United States and The Netherlands Women Health 1991 17 1 91 124 10.1300/J013v17n01_06 2048324

[B10] Macintyre S Hunt K Sweeting H Gender differences in health: are things really as simple as they seem? Soc Sci Med 1996 42 4 617 624 10.1016/0277-9536(95)00335-5 8643986

[B11] Luce PJB Addressing the women’s needs.2nd Annual Veteran Symposium. University of Louisville. Louisville ,KY.22-23 Feb 2010

[B12] Ladwig KH Marten-Mittag B Formanek B Dammann G Gender differences of symptom reporting and medical health care utilization in the German population Eur J Epidemiol 2000 16 6 511 518 10.1023/A:1007629920752 11049093

[B13] Parslow R Jorm A Christensen H Jacomb P Rodgers B Gender differences in factors affecting use of health services: an analysis of a community study of middle-aged and older Australians Soc Sci Med 2004 59 10 2121 2129 10.1016/j.socscimed.2004.03.018 15351477

[B14] Dunlop S Coyte PC McIsaac W Socio-economic status and the utilisation of physicians' services: results from the Canadian National Population Health Survey Soc Sci Med 2000 51 1 123 133 10.1016/S0277-9536(99)00424-4 10817475

[B15] Mueller KJ Patil K Boilesen E The role of uninsurance and race in healthcare utilization by rural minorities Health Serv Res 1998 33 3 Pt 1 597 610 9685124 PMC1070278

[B16] Hoff RA Rosenheck RA Female veterans' use of Department of Veterans Affairs health care services Med Care 1998 36 7 1114 1119 10.1097/00005650-199807000-00017 9674628

[B17] Hoff RA Rosenheck RA Utilization of mental health services by women in a male-dominated environment: the VA experience Psychiatr Serv 1997 48 11 1408 1414 9355167 10.1176/ps.48.11.1408

[B18] Burgess JF Jr DeFiore DA The effect of distance to VA facilities on the choice and level of utilization of VA outpatient services Soc Sci Med 1994 39 1 95 104 10.1016/0277-9536(94)90169-4 8066492

[B19] LaVela SL Smith B Weaver FM Miskevics SA Geographical proximity and health care utilization in veterans with SCI&D in the USA Soc Sci Med 2004 59 11 2387 2399 10.1016/j.socscimed.2004.06.033 15450711

[B20] Outpatient Health Care Copay (VA Health Care Eligibility & Enrollment) http://www4.va.gov/healtheligibility/costs/OutpatientCopay.asp

[B21] Asada YKG Equity in health services use and intensity of use in Canada. BMC Health Services Research BMC Health Services Research 2007 7 41 10.1186/1472-6963-7-41 17349059 PMC1829158

[B22] Duan N Manning WG Jr Morris CN Newhouse JP A Comparison of Alternative Models for the Demand for Medical Care Journal of Business & Economic Statistics 1983 1 2 115 126 10.2307/1391852

[B23] Rosenheck R Massari L Wartime military service and utilization of VA health care services Mil Med 1993 158 4 223 228 8479627

[B24] Wolinsky FD Coe RM Mosely RR Homan SM Veterans' and nonveterans' use of health services. A comparative analysis Med Care 1985 23 12 1358 1371 10.1097/00005650-198512000-00005 3910973

[B25] Maciejewski ML Perkins M Li YF Chapko M Fortney JC Liu CF Utilization and expenditures of veterans obtaining primary care in community clinics and VA medical centers: an observational cohort study BMC Health Services Research 2007 7 56 10.1186/1472-6963-7-56 17442115 PMC1855054

[B26] Hall JA Dornan MC Patient sociodemographic characteristics as predictors of satisfaction with medical care: a meta-analysis Soc Sci Med 1990 30 7 811 818 10.1016/0277-9536(90)90205-7 2138357

[B27] Home and Community Based Services (HCBS) http://www.hcbs.org/moreInfo.php/doc/2692

[B28] Pierce PF Antonakos C Deroba BA Health care utilization and satisfaction concerning gender-specific health problems among military women Mil Med 1999 164 2 98 102 10050564

[B29] http://www.allacademic.com http://www.allacademic.com/meta/p_mla_apa_research_citation/0/9/3/3/8/p93380_index.html

[B30] Vogt D Bergeron A Salgado D Daley J Ouimette P Wolfe J Barriers to Veterans Health Administration care in a nationally representative sample of women veterans J Gen Intern Med 2006 21 Suppl 3 S19 25 10.1111/j.1525-1497.2006.00370.x 16637940 PMC1513162

[B31] United States Congress House Committee on Veterans' Affairs Subcommittee on Oversight and Investigations VA provision of health care to women veterans and related issues One Hundred Third Congress 1993 64/3 first 103 119

[B32] Yano EM Goldzweig C Canelo I Washington DL Diffusion of innovation in women's health care delivery: the Department of Veterans Affairs' adoption of women's health clinics Womens Health Issues 2006 16 5 226 235 10.1016/j.whi.2006.07.002 17055375

[B33] Washington DL Yano EM Simon B Sun S To use or not to use. What influences why women veterans choose VA health care Journal of General Internal Medicine 2006 21 Suppl 3 S11 8 10.1111/j.1525-1497.2006.00369.x 16637939 PMC1513176

[B34] http://www.visn21.va.gov http://www.visn21.va.gov/VISN21/docs/VHM/HealthMattersVol1_2008.doc

[B35] Hoge CW Castro CA Messer SC McGurk D Cotting DI Koffman RL Combat duty in Iraq and Afghanistan, mental health problems, and barriers to care N Engl J Med 2004 351 1 13 22 10.1056/NEJMoa040603 15229303

[B36] Carney CP Sampson TR Voelker M Woolson R Thorne P Doebbeling BN Women in the Gulf War: combat experience, exposures, and subsequent health care use Mil Med 2003 168 8 654 661 12943043

[B37] Nice DS Hilton S Sex differences and occupational influences on health care utilization aboard US Navy Ships Military Psychology 1994 6 2 109 123 10.1207/s15327876mp0602_3

[B38] McNeil M Hayes P Women's health care in the VA system: another "patchwork quilt" Womens Health Issues 2003 13 2 47 49 10.1016/S1049-3867(03)00025-2 12732439

[B39] Laine C Davidoff F Lewis CE Nelson EC Nelson E Kessler RC Delbanco TL Important Elements of Outpatient Care: A Comparison of Patients' and Physicians' Opinions Ann Intern Med 1996 125 8 640 645 8849148 10.7326/0003-4819-125-8-199610150-00003

